# Imaging Techniques for the Study of Fibrosis in Atrial Fibrillation Ablation: From Molecular Mechanisms to Therapeutical Perspectives

**DOI:** 10.3390/jcm10112277

**Published:** 2021-05-24

**Authors:** Francesco De Sensi, Diego Penela, David Soto-Iglesias, Antonio Berruezo, Ugo Limbruno

**Affiliations:** 1 Cardiology Department, Misericordia Hospital, ESTAV Toscana Sud Est, Via Senese 161, 58100 Grosseto, Italy; ulimbru@tin.it; 2 Teknon Medical Center, Heart Institute, Calle Vilana 12, 08022 Barcelona, Spain; dpenela30@gmail.com (D.P.); sotin1985@gmail.com (D.S.-I.); antonio.berruezo@quironsalud.es (A.B.)

**Keywords:** atrial fibrillation, atrial fibrosis, atrial remodeling, atrial cardiomyopathies, atrial failure, cardiac magnetic resonance, multi detector computed tomography, left atrial strain

## Abstract

Atrial fibrillation (AF) is the most prevalent form of cardiac arrhythmia. It is often related to diverse pathological conditions affecting the atria and leading to remodeling processes including collagen accumulation, fatty infiltration, and amyloid deposition. All these events generate atrial fibrosis, which contribute to beget AF. In this scenario, cardiac imaging appears as a promising noninvasive tool for monitoring the presence and degree of LA fibrosis and remodeling. The aim of this review is to comprehensively examine the bench mechanisms of atrial fibrosis moving, then to describe the principal imaging techniques that characterize it, such as cardiac magnetic resonance (CMR) and multidetector cardiac computed tomography (MDCT), in order to tailor atrial fibrillation ablation to each individual.

## 1. Introduction

Atrial fibrillation (AF) is the most prevalent form of cardiac arrhythmia [1]. Its prevalence has increased over time and continues to rise in a pandemic manner. In fact, estimated AF cases in Europe are expected to move from 8.8 million in 2010 to 17.9 million in 2060 [2]. During the last 50 years, age-adjusted prevalence of AF quadrupled from 20.4 to 96.2 cases per 1000 person-years in men and from 13.7 to 49.4 cases per 1000 person-years in women [3]. The longer survival has increased the risk of age-related diseases such as atrial fibrillation and its related mortality. Indeed, although this arrhythmia is frequently asymptomatic, it is far from a benign condition because is associated with increased risk of stroke [4], heart failure [5], cognitive dysfunction [6], and cardiovascular or all-cause death [7]. An understanding of the underlying pathophysiological mechanisms is needed to implement preventive strategies for AF development, and improve medical and interventional treatment of this epidemic disease. The aim of this review is to comprehensively examine the bench mechanisms underpinning atrial fibrosis, then to describe the principal imaging techniques that characterize it, to tailor atrial fibrillation ablation for everyone. 

## 2. Atrial Fibrosis as Major Determinant of Atrial Fibrillation Genesis and Sustenance

Historically, AF has been classified into three patterns: paroxysmal, persistent, and permanent [8]. The first one is often not associated with cardiomyopathy and is triggered by pulmonary veins fires, or, in 10–20% of cases, by non-pulmonary ectopic foci, such as the left atrial appendage, coronary sinus, superior vena cava, and crista terminalis [9]. The other two forms are generally related to diverse pathological conditions affecting the atria, such as ischemic, valvular, genetic, and idiopathic cardiomyopathies [10,11]. All these conditions lead to remodeling processes, including collagen accumulation, fatty infiltration, and amyloid deposition. All these events generate atrial fibrosis, which contribute to AF [12].

Technically, fibrosis means the excessive deposition of the extracellular matrix (ECM), which is the fibrous tissue supporting muscular cells. When ECM exceeds, it alters myocytes milieu, losing the cardiac architectural integrity [13]. Different types of cardiac fibrosis have been characterized.

Histopathological classification divides fibrosis into two types:

(1) Replacement fibrosis: the process occurring after myocardial injury (as myocardial acute infarct) when necrotic cells are substituted by collagen and ECM. This is also called reparative fibrosis [14];
(2) Reactive fibrosis: an inflammatory process (mostly triggered by volume or pressure overload or genetic-mediated) of fibrous tissue deposition between cells (interstitial) and/or vessels (perivascular) causing disarray [15].

Depending on fibrotic tissue pattern, size, and distribution, we can describe fibrosis as [16]:

(1) Interstitial: widening and thickening of the ECM;
(2) Compact: consists of areas of dense collagen, deprived of any myocardial tissue;
(3) Diffuse: involves mixed areas of myocardial and collagen fibers;
(4) Patchy: consists of myocardial and collagen bundles and long strands.

Each type of pattern is not mutually exclusive. Different patterns and types of fibrosis may coexist in the same atrium. There are several clinical implications of these different types of fibrotic architecture on the related arrhythmogenesis. For example, compact fibrosis, although acutely severe, is less proarrhythmic than diffuse, patchy, and interstitial, and mainly promotes organized reentry (flutter) as circus movements around the fibrosis area due to unidirectional block [17]. On the contrary, patchy fibrosis is most prone to arrhythmogenesis because of development of zig-zag electrical conduction between the various bundles and long strands [18]. Diffuse fibrosis can, importantly, decrease the conduction velocity, leading to spiral wave formations, enhancing AF initiation and perpetuation [19]. Purely interstitial fibrosis, separating myocardial bundles, impairs transverse conduction without impacting longitudinal conduction and provoking anisotropic conduction [20]. In fact, the propagation perpendicular to the fiber direction becomes asynchronous, because activation must follow a tortuous route between the electrical barriers imposed by the collagen fibers. It has been shown that the presence of thick interstitial collagen strands is highly related to persistent AF [21].

## 3. Molecular Mechanisms

### 3.1. Cellular and Molecular Signaling of Fibrogenesis

**Figure 1:**
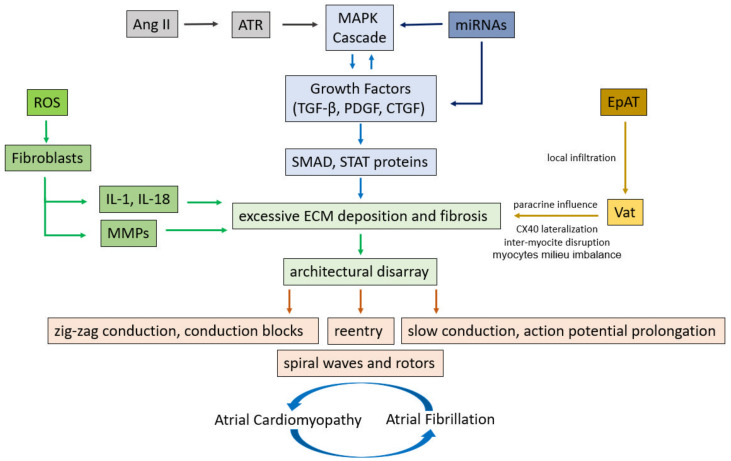
Cellular and molecular pathways involved in the structural and electrical remodeling leading to atrial fibrillation. Ang II = Angiotensin II, ATR = Angiotensin receptor, MAPK = mitogen-activated protein kinase, TGF-*β* = Transforming growth factor-*β*, PDGF = platelet derived growth factor, CTGF = connective tissue growth factor, SMAD = Small mothers against decapentaplegic, STAT = signal transducer and activator of transcription, ROS = reactive oxygen species, IL-1 = interleukin-1, IL-18 = Interleukin-18, MMPs = matrix metalloproteinases, ECM = extra cellular matrix, miRNAs = microRiboNucleicAcids, EpAT = Epicardial Adipose Tissue, VaT = Visceral adipose Tissue.

Several cellular and molecular pathways are involved in the fibrogenesis (Figure 1). Atrial fibroblasts, different from their ventricular counterpart, show a higher response to mitogenic factors, such as the platelet-derived growth factor (PDGF), angiotensin II (Ang II) and tissue growth factor (TGF-*β*-1) [22]. Angiotensin (AT) is one of the most important molecular mechanisms, promoting inflammatory chemokine expression in atrial fibroblasts, and inducing fibroblast proliferation by increasing the expression of profibrotic TGF-*β*-1 [23]. Moreover, AT-1 induces reactive oxygen species (ROS) activation and proinflammatory responses. ROS are involved in the profibrotic differentiation of fibroblasts into myofibroblasts regulating collagen synthesis and matrix metalloproteinase (MMPs) activity: the main enzymes of ECM degradation [24]. Recently, some translational studies identified the pivotal role of non-coding microRNAs (miRNAs) in cardiac fibrosis. For example, microRNA-30c suppresses the pro-fibrogenic effects of cardiac fibroblasts induced by TGF-*β*1 and prevents atrial fibrosis by targeting TGF*β*RII. Thus, a decreased expression of miRNA-30c favors ECM deposition [25]. Furthermore, cardiac fibroblast proliferation is modulated by micro-RNA-10a through the TGF-*β* 1/SMADs pathway. Small Mothers Against Decapentaplegic (SMADs) comprise a family of structurally similar proteins that are the main signal transducers for receptors of the transforming growth factor beta (TGF-*β*) superfamily and of the mitogen-activated protein kinase (MAPK) cascade, which are critically important for regulating cell development and growth [26].

The continuous evolution in the knowledge of all these interconnected cellular and molecular factors, promoting atrial fibrosis, is a promising field in the understanding of mechanisms, characterization of their pathophysiology, and development of therapeutic perspectives in atrial fibrillation.

### 3.2. Adipose Tissue as a Novel Risk Factor of Fibrosis and Atrial Fibrillation

It is well known that an increased body mass index (BMI) often coexists with several cardiovascular (CV) risk factors, such as arterial hypertension, insulin resistance and low-grade inflammation. Morbidity and mortality in these patients is sensibly amplified [27]. The prevalence of obesity is escalating alarmingly, and the number of affected people follows an exponential growth curve [28]. Data from the Framingham Heart Study clearly demonstrated the association between obesity and AF risk over a 14-year observation period, with a 4–5% increase in AF risk for every unit increase in BMI [29]. Several factors contribute to explaining the increased risk of AF in patients with obesity and metabolic syndrome. These include changes in volume status, cardiac loading, energy substrate utilization, tissue metabolism and systemic inflammation. Obese patients display a significant increase in the left atrial size and pressure, which is a potent predictor of AF occurrence over 10-year follow-up [27]. The accumulation of both visceral (VaT) and epicardial adipose tissue (EpAT) leads to a condition of chronic low-grade inflammation, characterized by increased levels of profibrotic and pro-hypertrophic cytokines, such as TNF-*α*, iL-6 and iL-1*β* [30]. Besides atrial fibrosis and hypertrophy, fat-related inflammatory stimuli can disturb calcium homeostasis and channel function, mainly via the activation of Nad(P)H oxidase activity, nitric oxide synthase uncoupling, eventually leading to the accumulation of reactive oxygen species (ROS) [31]. Obesity and insulin resistance also lead to the activation of the renin-angiotensin-aldosterone system (RAAS), transforming growth factor beta (TGF-*β*), connective tissue growth factor (cTGF) and endothelin-1, all leading to interstitial collagen deposition and subsequent defects of atrial conduction due to a substrate favoring re-entry and AF perpetuation [32]. Moreover, recent studies suggest that epicardial fat—which strongly correlates with visceral fat—is actively involved in the secretion of several proinflammatory cytokines which contribute to adverse left atrial remodeling, AF development and poor AF-related outcome [33]. In fact, local epicardial adipose tissue (EpAT) depots may contiguously infiltrate the atrial myocardium, leading to architectural disarray. This phenomenon may determine action potential prolongation and conduction slowing. Furthermore, EpAT is endocrinologically active in protein synthesis and release (paracrine effect), causing fibrosis and the lateralization of connexin-40, with inter-myocite disruption losing the regular architecture (Figure 1). This finally results in aberrant excitability and conduction heterogeneity and, thus, atrial fibrillation [34]. Important therapeutic perspectives can be derived from this knowledge. For example, targeting the EpAT adipokine population with antibody therapies may reduce the large inflammatory component decrementing AF genesis and maintenance. Indeed, reducing growth factors and profibrotic cytokines levels as well as local oxidative stress may influence the milieu that increases AF risk [35].

### 3.3. From Atrial Fibrosis to Atrial Cardiomyopathy

The atrium has always been the neglected heart structure in favor of its counterpart, the ventricle. However, in recent years, awareness of atrial pathologies, which may have a substantial impact on cardiac performance, arrhythmia occurrence, and stroke risk, has been raised. In 2016, an intercontinental working group defined, for the first time, the term “atrial cardiomyopathy”, indicating “any complex of structural, architectural, contractile or electrophysiological changes affecting the atria with the potential to produce clinically relevant manifestations” [36]. They even defined a working histological/pathophysiological classification scheme using the acronym EHRAS (EHRA: European Heart Rhythm Society/HRS: Hear Rhythm society/APHRS: Asian Pacific Heart Rhythm Society/SOLAECE: Sociedad Latino Americana de Estimulacion Cardiaca y Electrofisiologia) recognizing four classes:

(1) Primarily cardiomyocytes changes (typical of Lone AF, genetic disease, and diabetes);
(2) Primarily fibroblast changes (due to ageing or smoking);
(3) Combined cardiomyocyte-fibroblast dependent fibrosis (present in valvular disease and chronic heart failure);
(4) Primarily non-collagen deposits (as in isolated atrial amyloidosis, granulomatosis, inflammatory infiltrates, glycosphingolipids).

Although this classification is purely descriptive, it has the aim of describing pathological changes in biopsies and correlating pathologies with results obtained from imaging technologies.

The term atrial remodeling is used to indicate the effective change in atrial wall thickness/characterization and/or atrial chamber size and shape [37]. There are several clinical scenarios in which left atrial remodeling predisposes the development of atrial cardiomyopathy (i.e.: hypertension, diabetes, myocarditis, ageing). Atrial remodeling is considered a marker for adverse cardiovascular outcomes, such as, for example, in atrial fibrillation and in patients with left ventricular diastolic or systolic dysfunction. Left atrial enlargement and dysfunction usually represents maladaptive structural remodeling, which has prognostic relevance in these patients [37]. Maladaptive remodeling often promotes electrical remodeling that, in turn, facilitates the occurrence of atrial fibrillation. Two main mechanisms accelerate left atrial remodeling develop, fast atrial arrhythmias and both, pressure, and volume overload. Left atrial remodeling can be classified into three singular entities, which are often interrelated:

(a) Structural remodeling, consequence of increases in interstitial fibrosis that result in atrial dilatation;
(b) Functional remodeling, characterized by a left atrial failure;
(c) Electrical remodeling that predisposes to atrial arrhythmias due to modifications to the substrate that promote reentry due to the heterogeneity of current conduction, shortening of action potentials, depolarization of resting cardiomyocytes, and induction of spontaneous phase 4 depolarization [38].

Presence of an isolated kind of remodeling is possible. However, often, the three forms coexist at the same time and enhance each other. There is rising interest in monitor left atrial remodeling in the clinical practice, with two main arguments:

(a) First, it is a potential reversible process. Modifying cardiovascular conditions, including hypertension or heart failure, can reverse the remodeling process, especially in the early stages of LA structural and functional remodeling. Moreover, some medical interventions leading to a reduction in the LA arrhythmic burden, for example, isolation of the pulmonary veins, have been shown to improve LA function [39]. Patients free of AF recurrence after catheter ablation can show a significant reduction in LA fibrosis burden in follow-up cardiac magnetic resonance (CMR) studies [40];
(b) LA remodeling is a prognostic factor. LA volume and function are related to the probability of developing atrial fibrillation [41] and are an independent prognostic marker in patients with heart failure and reduced ejection fraction [42]. Atrial fibrosis has been demonstrated to be associated with stroke and the presence of spontaneous echo contrast [43,44].

In this scenario, cardiac imaging appears to be a promising noninvasive tool for monitoring the presence and degree of LA remodeling. Moreover, imaging information can be incorporated into the clinical decision-making. Hypothetically, in the case of a severe atrial dilatation with extensive atrial fibrosis, it might not be justified to schedule the patients for an invasive approach as atrial fibrillation unless no other treatment options are available. On the other hand, even a patient with a longstanding AF can be a good candidate for ablation if imaging evidences a low degree of remodeling.

## 4. Evaluation of Fibrosis for Prognosis and Imaging to Guide Ablations

### 4.1. Late Gadolinium Enhancement Cardiac Magnetic Resonance (LGE-CMR)

In the last decade, CMR has emerged as a novel, fascinating imaging tool for evaluating LA remodeling. CMR image delivers a precise endocardial border definition, and its strength allows a complete measure of the LA atrial volume, rather than a simple area length. CMR is becoming more and more available and does not exposes patient to ionizing radiation. In fact, in recent years, CMR has become the gold standard for volumetric analysis. However, the more fascinating aspect of CMR is tissue characterization. LGE-CMR can identify, quantify, and characterize atrial fibrosis.

### 4.2. LGE-CMR Image Acquisition

LGE-CMR is considered a standard imaging technique to characterize atrial scarring. Gadolinium is used as a contrast agent to highlight areas of fibrosis which differentiate healthy tissue (with high gadolinium washout velocity) from scarred tissue (slower washout). A good quality of the acquired images is dependent on heart movements and breathing. Therefore, multiple images during consecutive breath holds are recorded, obtaining multiple short axis planes along the axis of the left atrium. Due to the high chances of recording artifacts which may impair the reconstruction of the scar, 2 and 3-dimensional acquisition protocols have been developed, based on 2- and 3D navigator-gated inversion recovery sequences, avoiding slice-shifting artifacts [45]. Moreover, the acquisition is usually electrocardiogram (ECG)-triggered to acquire images in the same moment of the cardiac cycle. Data acquisition is performed in atrial diastole to minimize motion artefact and extend data acquisition duration. Due to the irregularity of the cardiac cycle, evaluating atrial fibrosis during atrial fibrillation remains a challenge. For this reason, an external cardioversion is usually performed if irregular rhythms are present. Irregular respiratory pattern prolongs acquisition time and was also recognize as a frequent cause of poor image quality [46]. It was estimated that an appropriate image for fibrosis characterization can be obtained in 90% of CMR [47].

### 4.3. CMR Left Atrial Fibrosis Characterization

**Figure 2:**
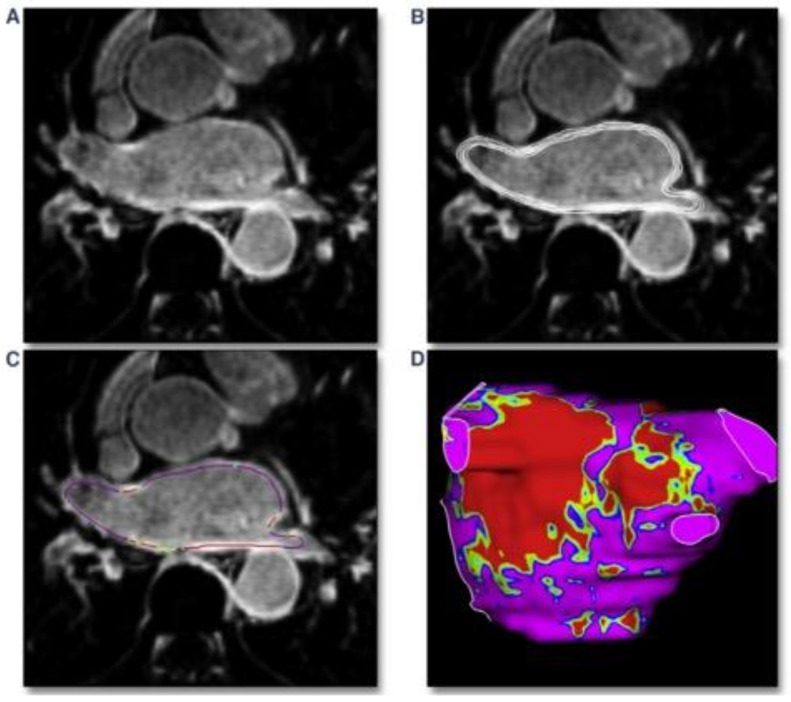
Post-processing phase of left atrium detected with cardiac MRI basing on pixel signal intensity. (**A**): MRI-LGE scan of the left atrium. (**B**): manual segmentation of the atrial wall with analysis of multiple layers from endocardium to epicardium. (**C**): pixel intensity map creation along the atrial wall. (**D**): 3-dimensional shell of the left atrium with pixel intensity map based on different colors; red: dense scar, green/yellow: border zone, purple: healthy tissue. Requested permission from [49].

Following the acquisition of LGE-CMR scan, images should be post-processed based on the identified pixel signal intensities (PSI). Endocardial and epicardial borders are usually semiautomatically demarcated for segmentation (Figure 2) by using the left atrial blood pool as a reference to identify enhanced LA pixels [48].

**Figure 3:**
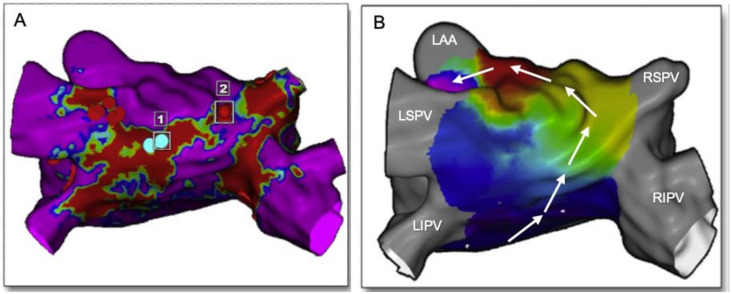
Usefulness of cardiac MRI in redo procedures of atrial fibrillation and atrial flutter ablation. (**A**): MRI-LGE of the left atrium in a patient with previous ablation of the roof presenting with atypical flutter. Red areas represent scar due to previous ablation (blue dots = 1). In point 2 (red dot), there is a portion of healthy tissue (purple) between the roof line and the RSPV. (**B**): Activation mapping during flutter with CARTO system depicts a circuit directed upwards along the posterior wall and crossing the gap identified at the MRI (white arrows). One-shot ablation in point 2 interrupted the roof-dependent atrial flutter. LAA: left atrial appendage, LSPV: left superior pulmonary vein, LIPV: left inferior pulmonary vein, RSPV: right superior pulmonary vein, RIPV: right inferior pulmonary vein. Modified from [49].

LA fibrosis distribution and shape are usually visualized using a 3D shell of the left atrium, applying a color code based on the PSI to distinguish healthy myocardium from scaring areas. As a result of the LA tissue characterization process, quantitative and qualitative information on the LA fibrosis is available. For a quantitative analysis of the scar, data are usually presented in grams of fibrosis or, alternatively, in volume/area percentage. The group of Marrouche use the Utah stage classification for a quantitative analysis of the fibrosis, based on LA wall enhancement, as a percentage of the total LA wall: stage I, defined as <10%, stage II >10 to <20%, stage III >20 to <30%, and stage IV >30% (Table 1). A quantitative analysis of the LA fibrosis has been shown to be related to the response to AF catheter ablation, independently of the presence of other comorbidities or AF behavior [50]. Moreover, the potential regression of structural alterations is possible when ablation is performed in early Utah stages [51]. The initial data also suggest that a decision-making approach for the selection of patients for AF ablation can be made based on the quantification of fibrosis [52]. A quantitative analysis of the fibrosis was also related to hard clinical outcomes, such as stroke [43]. The qualitative analysis of fibrosis (distribution and shape) can be used to plan AF intervention, since 3D pixel intensity maps can be imported into the navigator system to help in catheter ablation procedures. Bisbal and colleagues showed that LGE-CMR-delivered maps can successfully guide repeated PVI procedures by accurately identifying and localizing gaps and may reduce the procedural duration and radiofrequency application time [49] (Figure 3). A further hypothesis proposed for some groups is that areas of fibrosis identified in the LGE-CMR could become a target for ablation. In the recently published ALICIA trial, patients were randomized to receive pulmonary vein isolation (PVI) plus CMR-guided fibrosis ablation vs. PVI alone. After 12 months follow-up, there were no differences between groups in the recurrence rate. The results of the ongoing delayed-enhancement MRI Determinant of Successful Radiofrequency Catheter Ablation of Atrial Fibrillation (DECAAF II, ClinicalTrials.gov Identifier: NCT02529319) trial might shed light on the role of atrial fibrosis as a potential target for catheter ablation of AF.

### 4.4. Multidetector Cardiac Computed Tomography (MDCT)

**Figure 4:**
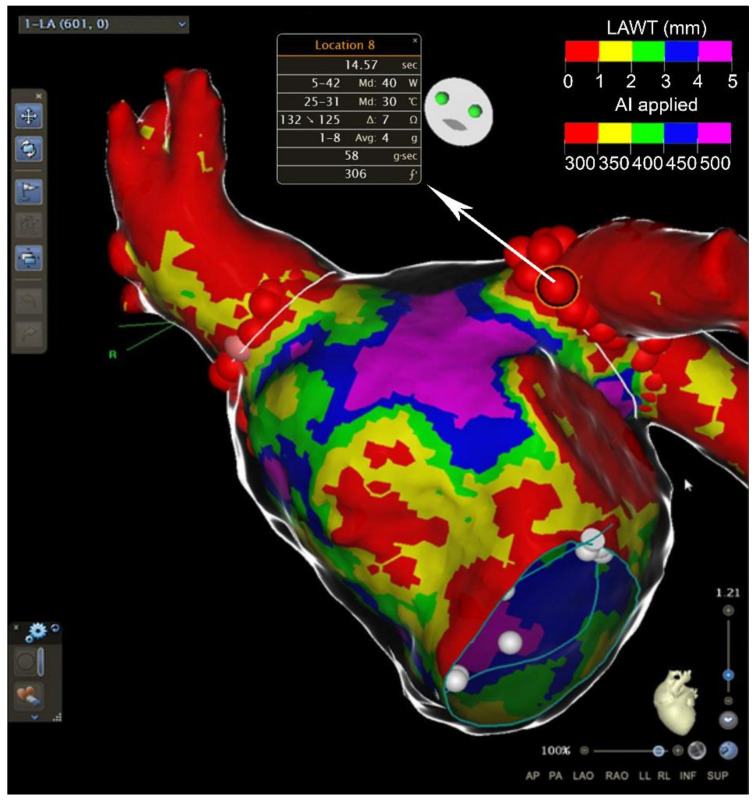
Usefulness of cardiac CT in atrial fibrillation ablation. Anterior view of the left atrium after atrial fibrillation ablation around the pulmonary veins. Atrial wall thickness (in mm) is depicted with different colors on the map. Major values in radiofrequency applications (AI) are used in thicker areas. AI = ablation index. LAWT = left atrial wall thickness.

MDCT has a greater spatial resolution (0.5 mm) compared with LGE-CMR, better defining the cardiac anatomy. On the other hand, it has a low contrast-to-noise ratio, a feature that reduces its ability to distinguish between normal myocardium and scar. Cardiac CT estimates LA volume without geometric assumptions, and thereby provides a more accurate evaluation of LA volume compared to TTE [53]. It can accurately define the course of the pulmonary veins, the atrial appendage location and morphology [54]. Moreover, MDCT permits the identification of sensitive extracardiac structures, i.e., the left phrenic nerve. As a consequence, MDTC is of great value for evaluation prior to pulmonary vein isolation for AF ablation. Moreover, due to its great spatial resolution, MDCT is able to accurately measure the left atrial wall thickness. The left atrial wall is a thin structure with heterogeneous thickness ranging from <1 mm to >5 mm. AF ablation durability can be limited by its inability to create transmural lesions in certain anatomical sites where left atrial wall thickness (LAWT) is higher [55]. In fact, it is an independent predictor of reconnection and AF recurrence after 12 months follow-up [56,57]. Berruezo and colleagues proposed a new approach for guiding pulmonary vein isolation by the LAWT. They recently report the feasibility of incorporating 3D LAWT maps into the navigation system, allowing a direct estimation of the WT at any point of the left atrium during the procedure [58]. They tailor radiofrequency energy, also delivering an ablation line design depending on the LAWT (Figure 4). This new approach to AF ablation can potentially increase the efficiency and safety of the ablation procedure. MDCT can also accurately identify and quantify epicardial adipose tissue (EpAT). It is, as previously cited, a potentially important player for atrial remodeling [35]. MDCT not only allows the identification of the EpAT location, it also allows a volumetric quantification defining the attenuation value range (usually −195 to −45 HU) for EAT segmentation. A recent study shows that regional EpAT affects local myocardial electrophysiology by direct infiltration in intermyocyte disruption, tissue fibrosis and gap junction remodeling [34]. It seems that the EpAT volume is correlated with a local slower activation time and an increased number of complex local potentials. A concordance could exist between the location of the fat and the electrical properties of the mapping during the ablation (slower conduction speed, fragmentation). Indeed, the properties of the fat could influence the risk of recurrence after ablation. However, further studies are needed to integrate this instrument in clinical practice.

### 4.5. Echocardiographic Strain

Left atrial strain measures local deformation of the myocardium and have been used to evaluate atrial function in various disease states. It has been shown that alteration in atrial function, measured by strain, precede atrial dilatation [59,60]. Therefore, left atrial strain emerged as a potential tool for detecting subclinical atrial dysfunction. Indeed, LA strain can be used as a subrogate of left atrial fibrosis. A previous study including 148 patients undergoing AF ablation showed that more than 60% of patients exhibited LA reverse remodeling after catheter ablation, and baseline LA strain, as an indirect marker of fibrosis, was an independent predictor of LA reverse remodeling [61]. It has been shown that the LA strain measured in patients with paroxysmal AF can predict progression to persistent AF, as well as atrial fibrillation recurrence after catheter ablation [62]. Yasuda and colleagues previously reported a series of 100 patients undergoing AF ablation [63]. The baseline LA strain was the most useful parameter in the prediction of AF recurrence, with a 0.84 area under the curve. Finally, LA strain has recently been shown to identify patients at high risk of AF among those that suffered from cryptogenic stroke, suggesting that echocardiographic quantification of LA remodeling has the potential for secondary prevention in this population [64].

## 5. Atrial Substrate Characterization and Its Implications for Clinical Management of the Patient with AF

The complexity of the mechanisms leading to atrial remodelling makes each AF patient very different. Thus, AF must be interpreted as the same electrocardiografic manifestation of a protean multifaced clinical syndrome. Its treatment must be considered beyond rhythm and/or rate control and the dichotomy of anticoagulate or not anticoagulate in an AF patient. In fact, a holistic management of AF must also comprise the identification of concomitant cardiovascular risk factors and comorbidity. In this sense, the renowned classification based on AF episode duration and temporal patterns (first episode, paroxysmal, persistent, permanent) presents some limitations. A new multidimensional AF classification must be sought, addressing specific domains with treatment and prognostic implications. For this reason, the new 4S-AF scheme proposed by the 2020 international guidelines is a characterization (more than a classification) based on pathophysiology. It includes four AF- and patient-related domains: Stroke risk, Symptoms, Severity of AF burden, and Substrate severity [65]. Concerning this last point, cardiac imaging undoubtedly plays a pivotal role in estimating the grade of atrial disease and could be included in the AF management decision-making process. It represents a paradigm shift in the integrated care approach which comprises five main principal actions in the CC to ABC pathway (Atrial fibrillation Better Care):

(1) C = confirm AF (with ECG recordings, cardiac devices or mobile health technology);
(2) C = characterize AF (with the 4S-AF scheme);
  to
(3) A = avoiding stroke (with anticoagulation or left atrial appendage closure);
(4) B = better symptoms (with rate or rhythm control);
(5) C = Cardiovascular risk and Comorbidity optimization (with aggressive risk factors interventions).

Compliance with such a management scheme has shown to improve population-based clinical outcomes in several nationwide AF cohorts [66,67]. Furthermore, using mobile health technology in the mobile Atrial Fibrillation App-II trial Guo and colleagues demonstrated the integrated holistic ABC approach, applied on an adult population of 1261 subjects (mean age 67.0 years), could reduce the composite outcome of ischaemic stroke/systemic thromboembolism, death, and rehospitalization [68].

## 6. Future Perspectives and New Research Areas

While progress has been made in atrial fibrosis etiopathology and characterization, major gaps persist, which need further research, especially in the field of cardiac imaging and its potential role in the management of patient with AF, particularly those undergoing ablation. In fact, contemporary techniques do not allow:

(1) Explanation of the single (or multiple) arrhythmia mechanisms in each patient. Often, also during the ablative procedure, it is not possible to discern between ectopic (triggered) activity and/or re-entry. Furthermore, before the procedure, it is not possible to define AF as pulmonary vein (PV)-dependent vs. non-PV-dependent. New technologies, including integration between imaging and ECG and using artificial intelligence, are promising in this sense [69];
(2) Complete understanding of the prevalent molecular pathway leading to atrial fibrosis. In fact, if, in the future, it becomes possible to identify each mechanism in each patient, new therapeutical techniques could be applied to develop a specific therapy targeting that pathway (i.e., genic therapy) [70];
(3) Possible identification of a prevalent etiology of the AF (atrial fibrosis, circadian rhythm, ischaemia, autonomic imbalance, inflammation, and adiposity). Therefore, we have to manage all these factors together, without specificity. Functional methodologies studying atria innervation and perfusion are welcome in this field;
(4) Individuation of a pre-state of AF to carry out a primary prevention of AF development (and its related complication as heart failure and stroke), which, at present, is left to clinical discretion. Studying a score system including clinical and imaging factors to identify people with higher chances of developing AF could be useful. Whether this is reflected by improved atrial fibrosis needs further study.

## 7. Conclusions

In recent years, awareness of atrial cardiomyopathies has raised. Atrial remodeling is a complex phenomenon that impacts the atrial wall and chamber, including collagen accumulation, fibrosis formation and fatty infiltration. All these conditions have a substantial impact on cardiac performance, arrhythmia occurrence, and stroke risk. Atrial fibrosis characterization, with imaging methods such as CMR, MDTC and echography, can be useful to choose the appropriate patient undergoing AF ablation and to tailor the approach to each individual.

## Figures and Tables

**Table 1:** Utah stage classification of left atrial fibrosis with cardiac MRI and correlation with outcomes after atrial fibrillation ablation in the DECAAF study.

Stage	% of Fibrosis at MRI	1-year Recurrences Rate after Ablation
I	<10% of AW	15.3%
II	10 > 20% of AW	32.6%
III	20 > 30% of AW	45.9%
IV	>30% of AW	51.4%

AW = atrial wall.
